# Extreme temperature and humidity exposure elevates acute intracerebral hemorrhage risk

**DOI:** 10.1016/j.isci.2025.113956

**Published:** 2025-11-05

**Authors:** Shengli Hu, Hao Peng, Yong Xu, Jun Zhao, Zhizhen Wei, Anwei Zhang, Can Huang, Yuting Si, Yingying Tang, Kuanming Huang

**Affiliations:** 1 Department of Cerebrovascular Disease Diagnosis and Treatment Center, Hubei Provincial Clinical Research Center of Central Nervous System Repair and Functional Reconstruction, Taihe Hospital, Hubei University of Medicine, Shiyan 442000, Hubei, China; 2 Department of Neurorehabilitation Centers, Hubei Provincial Clinical Research Center of Central Nervous System Repair and Functional Reconstruction, Taihe Hospital, Hubei University of Medicine, Shiyan 442000, Hubei, China; 3 School of Public Health, Hubei University of Medicine, Shiyan 442000, Hubei, China

**Keywords:** climatology, neuroscience

## Abstract

This study examines the association between extreme temperature, relative humidity, and acute intracerebral hemorrhage (ICH) risk using a time-stratified case-crossover design. Data from 2,284 ICH patients in the northwestern region of Hubei Province, China, were analyzed, focusing on extreme environmental conditions. Extreme low temperatures significantly increased ICH risk, peaking around 18 h post exposure, while low humidity was associated with a risk peak at 7 h post exposure. Sensitivity analyses confirmed the robustness of these findings, with extended lag periods showing sustained risk but decreasing magnitude over time. Subgroup analyses revealed higher vulnerability among lobar hemorrhage patients and older adults. These findings highlight the importance of environmental factors, particularly cold and low humidity, in ICH risk and stress the need for targeted public health interventions for vulnerable populations during extreme weather events.

## Highlights

• Time-stratified case-crossover analysis of ICH risk under extreme weather
• Cold exposure raised ICH risk, peaking 18 h after exposure
• Low humidity had a transient peak effect at 7 h post-exposure
• Deep hemorrhage patients showed greater environmental vulnerability

## Graphical abstract

**Figure:**
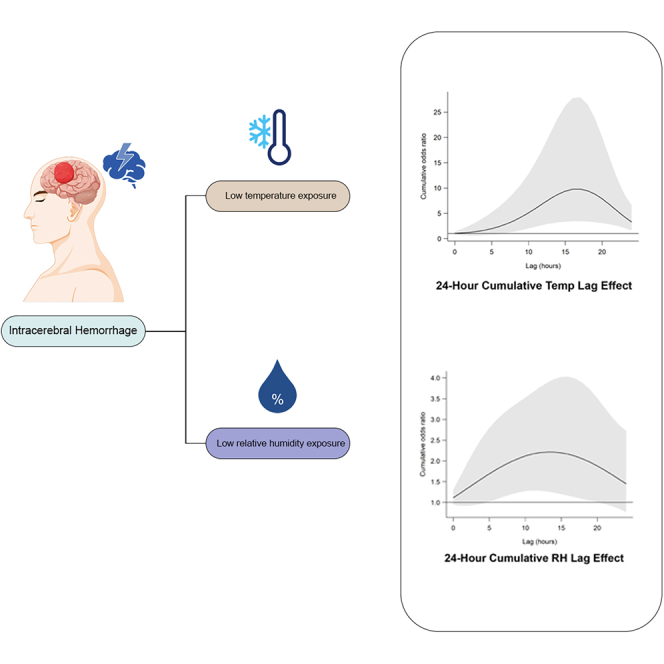

## Introduction

Stroke is recognized as a major public health burden globally—not only due to its persistently high incidence but also because it results in a substantial number of deaths and long-term disabilities. Numerous international studies have demonstrated that environmental factors such as ambient temperature, humidity, atmospheric pressure, extreme weather events, and air pollution are closely associated with stroke risk.^1^^,^^2^^,^^3^ Global data indicate that extreme temperatures can increase stroke risk via mechanisms such as sympathetic activation, blood pressure fluctuations, altered blood viscosity, and inflammation.^4^^,^^5^^,^^6^^,^^7^ Moreover, these effects often manifest in both the short-term and with a lag period.^8^^,^^9^

Intracerebral hemorrhage (ICH) accounts for 10%–20% of all stroke types; however, it is the most fatal subtype, with short-term mortality reaching approximately 40%–50%.^10^ Current research on the relationship between ambient temperature and stroke subtypes mainly focuses on ischemic stroke, and less attention is paid to hemorrhagic stroke. Exploring the relationship between ambient temperature and the onset of ICH is of great importance. Several studies have reported low temperatures were associated with an increased risk of intracranial hemorrhage.^11^^,^^12^^,^^13^

**Figure 1:**
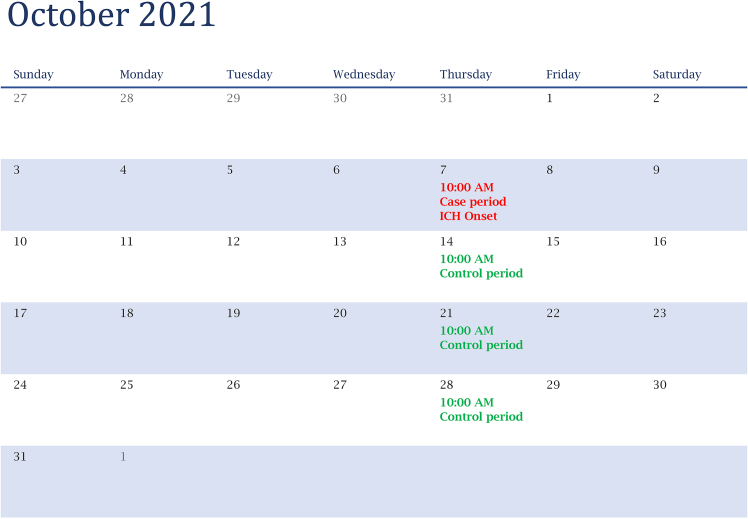
Diagram illustrating the case and control periods in a time-stratified case-crossover design Control periods were selected to match the same weekday within the same month as the ICH event.

Previous studies have examined the relationship between meteorological factors and ICH using diverse designs and datasets. Liu et al.^12^ conducted a Tianjin-based study (2014–2020) applying a time-stratified case-crossover approach with daily mean temperature and admission records, focusing on broad associations between temperature and ICH occurrence. Chen et al.^11^ analyzed a large Taiwanese health-claims dataset (2011–2020) to assess daily averages of temperature and atmospheric pressure, alongside 24-h changes, without incorporating hour-specific onset times. Herweh et al.^14^ used a global multicenter clinical trial database on hypertensive ICH in specific anatomic locations; however, the use of RCT-based eligibility criteria may have excluded more severe or diverse cases and did not adopt a case-crossover design. Previous studies offer valuable insights, but gaps remain in understanding hour-specific onset patterns, short-term lag effects, and combined temperature and humidity impacts. This study examines the immediate (0–24 h) effects of extreme low temperature and low relative humidity on acute ICH onset using hourly data from multiple stroke centers with distributed lag non-linear model (DLNM) based on a case-crossover design (Figure 1).

In this study, we collected data from multiple centers in northwest Hubei province, with the aim of investigating the association between ambient temperature, as well as its fluctuations, and the onset of ICH. Additionally, we assessed possible association modifiers, such as age, gender, smoking, alcohol consumption, Glasgow Coma Scale (GCS), and ICH location.

## Results

### Descriptive results

After exclusions, a total of 2,284 patients with acute ICH from three stroke centers were included in the final analysis (Figure S1). The mean (standard deviation, SD) age of these patients was 60.1 (11.5) years, with 1,378 males (60.3%) and 906 females (39.7%). Additionally, 34.3% were smokers and 44.3% reported alcohol consumption. Among these patients, the majority exhibited deep hemorrhage (73.2%), 39.2% presented with severe impairment of consciousness (GCS ≤8), and 26.9% experienced onset during the 18:00–23:59 time interval (Table 1). During the study period, the mean temperature (SD) from lag 0 to 24 h was 15.5 (8.6) °C, and the mean relative humidity (SD) was 72.2 (15.3) %. Pollutant levels exhibited significant variation, with a median PM_2.5_ concentration of 17.8 μg/m^3^ (interquartile range: 10.6 to 27.0 μg/m^3^) and a median O_3_ concentration of 79.1 μg/m^3^ (63.4–96.8 μg/m^3^) (Table 2).

**Table 1:** Summary of the associations between temperature (°C), relative humidity (%), and patient characteristics of acute intracerebral hemorrhage

Characteristic	Case, no. (%)	Temperature				Relative humidity			
		P_1_	P_99_	OR (95% CI)	*p* value	P_1_	P_99_	OR (95% CI)	*p* value
**Overall**	**2,284 (100.0)**	**−0.3**	**34.3**	**5.42 (2.49–11.78)**	NA	**23**	**100**	**1.43 (1.05–1.94)**	NA
Age, y									
18-60	1,154 (50.5)	−0.5	34.2	6.21 (2.00–19.31)	**Ref.**	22	100	1.41 (0.89–2.23)	**Ref.**
>60	1,130 (49.5)	−0.6	34.4	4.96 (1.54–15.95)	**0.786**	21	100	1.47 (0.92–2.35)	**0.895**
Gender									
Male	1,378 (60.3)	−0.7	34.4	6.00 (2.11–17.07)	**Ref.**	21	100	1.46 (0.94–2.27)	**Ref.**
Female	906 (39.7)	−0.2	34.0	4.48 (1.31–15.36)	**0.725**	21	100	1.47 (0.88–2.46)	**0.989**
Smoking									
No	1,501 (65.7)	−0.6	34.2	5.24 (1.95–14.07)	**Ref.**	21	100	1.56 (1.03–2.34)	**Ref.**
Yes	783 (34.3)	−0.6	34.4	6.21 (1.50–25.63)	**0.848**	21	100	1.36 (0.76–2.58)	**0.716**
Alcohol drinking									
No	1,272 (55.7)	−0.6	34.2	3.97 (1.38–11.44)	**Ref.**	21	100	1.49 (0.956–2.31)	**Ref.**
Yes	1,012 (44.3)	−0.4	34.4	9.18 (2.74–30.77)	**0.307**	21	100	1.38 (0.825–2.30)	**0.825**
GCS									
≤8	896 (39.2)	−0.6	34.4	3.79 (1.02–14.07)	**Ref.**	21	100	1.44 (0.84–2.47)	**Ref.**
9-11	304 (13.3)	−0.3	34.1	7.10 (0.810–62.25)	**0.627**	22	100	1.57 (0.66–3.74)	**0.867**
12-14	566 (24.8)	−0.6	33.2	6.49 (1.28–32.99)	**0.613**	21	100	1.31 (0.67–2.53)	**0.820**
15	518 (22.7)	−0.6	34.6	9.61 (1.77–52.27)	**0.394**	23	100	1.86 (0.93–3.70)	**0.572**
ICH location									
Deep	1,672 (73.2)	−0.4	34.2	6.25 (2.52–15.47)	**Ref.**	21	100	1.42 (0.97–2.10)	**Ref.**
Infratentorial	340 (14.9)	−0.2	33.7	3.11 (0.36–27.15)	**0.56**	20	100	1.05 (0.42–2.62)	**0.551**
Lobar	272 (11.9)	−0.9	34.5	3.80 (0.41–35.42)	**0.687**	22	100	2.52 (0.89–7.10)	**0.314**

Associations are presented as cumulative odds ratios comparing extreme low temperature (1st percentile) to reference temperature (99th percentile) over lag 0–12 h, and extreme low humidity (1st percentile) to reference humidity (99th percentile) over lag 0–6 h.

**Table 2:** Descriptive statistics of environmental data

Variables	Mean (SD)	Percentiles				
		1st	25th	50th	75th	99th
**Weather conditions**						

Temperature, °C	15.5 (8.6)	−0.2	8.3	15.1	23.2	31.1
Relative humidity, %	72.2 (15.3)	35.4	61.5	72.6	84.2	99.6

**Pollutants**						

PM_2.5_, μg/m^3^	21.7 (16.8)	3.5	10.6	17.8	27.0	76.2
PM_10_, μg/m^3^	41.4 (33.7)	5.4	21.0	33.8	51.9	151.8
SO_2_, μg/m^3^	7.7 (2.7)	2.8	5.5	7.5	9.7	14.2
NO_2_, μg/m^3^	9.1 (4.4)	2.0	5.7	8.0	11.7	23.2
O_3_, μg/m^3^	80.0 (27.0)	27.1	64.6	79.1	97.0	153.0
CO, mg/m^3^	0.6 (0.2)	0.2	0.5	0.6	0.7	1.3

CO, carbon monoxide; NO2, nitrogen dioxide; O3, ozone; PM2.5, fine particulate matter; PM10, coarse particulate matter; SO2, sulfur dioxide. The descriptive statistics of environmental data were calculated on the basis of moving backward 24 h from the onset hour of acute intracerebral hemorrhage.

The daily number of ICH cases, daily mean temperature, and daily mean relative humidity were analyzed using a 10-day moving average. The trend of ICH cases over time (Figure S2A) showed fluctuations, with noticeable peaks, while the temperature (Figure S2B) and humidity (Figure S2C) trends also varied, with temperature displaying more pronounced seasonality and relative humidity showing steadier fluctuations.

To assess the spatial representativeness of exposure assignments, we plotted the geographic distribution of patients’ residential addresses and corresponding interpolated temperature and humidity levels (Figure S3). This map illustrates that most patients lived within proximity to meteorological stations, suggesting minimal spatial misclassification of exposure estimates.

### Associations between low temperature and ICH onset

The 3D exposure-response surfaces for both extreme low temperature (Figure S4A) and relative humidity (Figure S4B) were plotted to examine the joint effects of temperature and humidity on ICH onset across different lag periods. For extreme low temperature, the odds ratio (OR) increased with longer lags, peaking at around 16–18 h, followed by a decline at later lags. This suggests that both temperature and lag interact to influence risk, with more pronounced effects at colder temperatures and certain lag windows. Similarly, for relative humidity, the surface indicated a strong association with ICH onset at lower humidity levels, which gradually decreased over time, reinforcing the observed pattern of a steady risk increase followed by a decline at longer lags.

**Figure 2:**
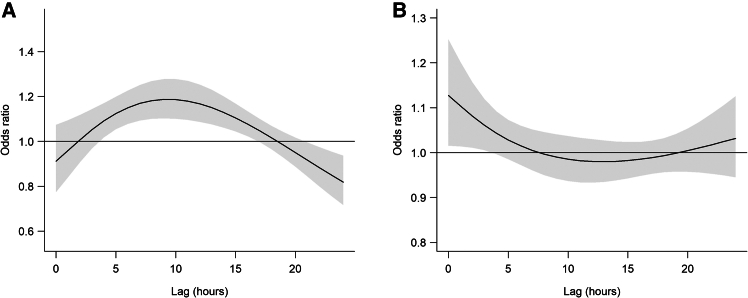
Lag–response patterns for acute intracerebral hemorrhage onset (A) Lag pattern for temperature. (B) Lag pattern for relative humidity. Solid lines represent odds ratios; grey shaded areas indicate 95% confidence intervals (0–24 h lag).

**Figure 3:**
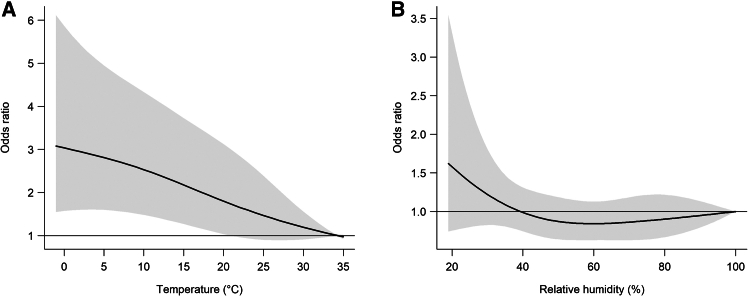
Overall exposure–response relationships for acute intracerebral hemorrhage risk (A) Relationship with ambient temperature. (B) Relationship with relative humidity. Solid lines represent odds ratios; grey shaded areas indicate 95% confidence intervals (0–24 h lag).

Figure 2A shows the lag patterns for ORs of ICH onset at extremely low temperatures compared with the reference temperatures. The risk increased with lag, peaking at lag 9–11 h (e.g., OR = 1.19 at lag11, 95% CI: 1.10–1.27), and gradually declined but remained statistically significant up to lag 16. Relative humidity showed a comparable pattern, with the strongest association observed at lag 0 h (OR = 1.13, 95% CI: 1.02–1.25), which gradually declined but remained statistically significant through lag 3 (OR = 1.06, 95% CI: 1.01–1.12) (Figure 2B). Subsequently, we plotted the cumulative exposure-response curves for the association between low temperature and ICH onset over lags 0 to 24 h (Figure 3A). The risk of ICH onset was monotonically increasing with lower temperatures and tended to level off at extremely high temperatures. A similar trend was observed for low humidity (Figure 3B), where ICH risk increased with decreasing humidity and plateaued above approximately 60%. In the cumulative time window with a lag of 0–16 h, the cumulative OR for extreme low temperatures was 5.42 (95% CI: 2.49–11.78).

**Figure 4:**
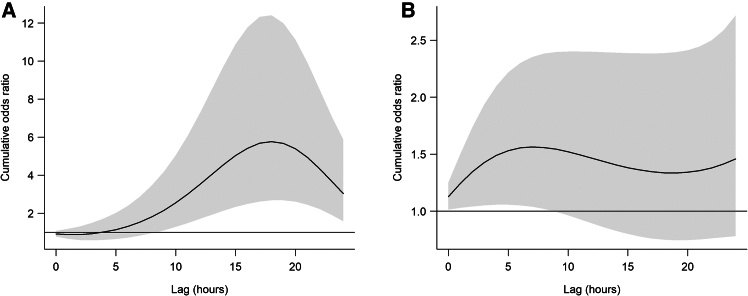
Cumulative effects on acute intracerebral hemorrhage onset over 24 hours (A) Cumulative effect of temperature. (B) Cumulative effect of relative humidity. Solid lines represent odds ratios; grey shaded areas indicate 95% confidence intervals.

As shown in the cumulative effect plot (Figure 4A), the risk gradually increased over time, peaked at 18 h (OR = 5.77, 95% CI: 2.68–12.41), and then decreased through to 24 h. This pattern suggests a “harvesting” effect, where the initial risk increase is followed by a reduction as vulnerable individuals experience the immediate impact, leading to fewer cases over time. Meanwhile, extreme low humidity (1st percentile relative humidity: 23%) was also significantly associated with ICH, with an OR of 1.43 (95% CI: 1.05–1.94), though its effect size was considerably smaller compared to low temperature (Table 1). Notably, Figure 4B demonstrates a “gradual rise and steady decline” pattern in the risk associated with humidity. The risk gradually increased from 0 h post exposure, peaking at a lag of 7 h (OR = 1.56, 95% CI: 1.04–2.35), followed by a gradual decline while remaining significant until a lag of 24 h. This also suggests a potential harvesting effect, where the peak of the risk is concentrated in the immediate period following exposure, after which the risk diminishes as the acute vulnerability stabilizes. To further clarify these time-sensitive patterns, Figure S5A presents the effect of extremely low temperature at a 16-h lag and low relative humidity at a 3-h lag (Figure S5B). The plot highlights that temperature demonstrates a more pronounced delayed effect, with the highest risk observed at around 16 h post exposure, whereas low humidity exhibits a quicker but still meaningful increase in ICH risk peaking near 4 h. These differential lag structures underscore the distinct physiological mechanisms by which cold and dryness may trigger cerebrovascular events. To assess potential interaction effects between temperature and humidity, we incorporated a temperature × humidity interaction term in the DLNM. The likelihood ratio test indicated no significant interaction (*p* = 0.999).

By comparing the extremely low temperatures with the reference, we computed ORs and 95% CIs of ICH. Table 1 summarizes the cumulative associations between extremely low temperature and ICH onset over lags 0–16 h, as well as the association between extremely low relative humidity and ICH onset over lags 0–3 h. In subgroup analyses (Table 1), exposure to extreme low temperature (1st percentile vs. 99th percentile) was significantly associated with increased odds of ICH overall (OR = 5.42, 95% CI: 2.49–11.78). The association was observed across all major subgroups, including younger adults aged 18–60 years (OR = 6.21, 95% CI: 2.00–19.31) and older adults aged ≥60 years (OR = 4.96, 95% CI: 1.54–15.95), males (OR = 6.00, 95% CI: 2.11–17.07) and females (OR = 4.48, 95% CI: 1.31–15.36), non-smokers (OR = 5.24, 95% CI: 1.95–14.07) and smokers (OR = 6.21, 95% CI: 1.50–25.63), as well as non-drinkers (OR = 3.97, 95% CI: 1.38–11.44) and drinkers (OR = 9.18, 95% CI: 2.74–30.77). None of the between-group differences reached statistical significance (all *p* for interaction >0.30).

Relative humidity also showed a significant overall association with ICH (OR = 1.43, 95% CI: 1.05–1.94). Among subgroups, non-smokers had a slightly higher estimated effect (OR = 1.56, 95% CI: 1.03–2.34) compared with smokers (OR = 1.36, 95% CI: 0.76–2.58), although the difference was not statistically significant (*p* for interaction = 0.716). No significant heterogeneity was found by age, gender, alcohol consumption, GCS category, or ICH location. Across ICH subtypes, patients with lobar hemorrhage exhibited a relatively higher OR for low humidity (OR = 2.52, 95% CI: 0.89–7.10), whereas the effect was smaller in deep (OR = 1.42, 95% CI: 0.97–2.10) and infratentorial hemorrhage (OR = 1.05, 95% CI: 0.42–2.62), although these effects were also not statistically significant (*p* = 0.314).

Overall, these results suggest that extreme cold and dry exposures elevate ICH risk broadly across demographic and clinical subgroups, with limited evidence of substantial effect modification.

The robustness of the primary associations was verified through a series of sensitivity analyses (Table 3). The link between extremely low temperature and ICH remained consistently significant across all model modifications. Increasing the degrees of freedom for the natural cubic spline from 3–4 had little impact on the association, with the OR only slightly attenuated to 4.38 (95% CI: 1.87–10.31). Extending the maximum lag period to 36 h (OR = 3.09, 95% CI: 1.45–6.58) and 48 h (OR = 2.74, 95% CI: 1.31–5.74) further reduced the effect estimates, suggesting a diminishing but still present risk beyond 24 h. Notably, when extreme values of temperature and humidity were retained in the model, the estimated temperature effect increased to OR = 5.73 (95% CI: 2.65–12.38), indicating that cold extremes may amplify ICH risk.

**Table 3:** Associations between temperature, relative humidity, and acute intracerebral hemorrhage across multiple sensitivity analyses

Variable^a^	OR (95% CI)	
	Temperature	Relative humidity
Main analysis^b^	5.42 (2.49–11.78)	1.43 (1.05–1.94)
**remove air pollutant covariates**	5.17 (2.47–10.81)	1.41 (1.05–1.88)

**Change model parameters**		

df of natural cubic spline: 4	4.38 (1.87–10.31)	1.55 (1.03–2.33)
Maximum lag period: 36 h	3.09 (1.45–6.58)	1.29 (0.98–1.69)
Maximum lag period: 48 h	2.74 (1.31–5.74)	1.22 (0.95–1.56)
Retaining extreme temperature and relative humidity values	5.73 (2.65–12.38)	1.21 (0.94–1.57)

**Various quantiles of low temperature and low relative humidity**		

2.5th percentile (temperature: 0.7°C, relative humidity: 28%)	5.31 (2.51–11.22)	1.33 (1.04–1.71)
5th percentile (temperature: 2.0°C, relative humidity: 34%)	5.19 (2.52–10.70)	1.23 (1.00–1.52)
10th percentile (temperature: 4.0°C, relative humidity: 41%)	4.96 (2.50–9.81)	1.14 (0.95–1.37)
15th percentile (temperature: 5.6°C, relative humidity: 47%)	4.71 (2.44–9.06)	1.08 (0.90–1.29)

a Associations are presented as cumulative odds ratios comparing extreme low temperature (1st percentile) to reference temperature (99th percentile) over lag 0–12 h, and extreme low humidity (1st percentile) to reference humidity (99th percentile) over lag 0–6 h.

b Settings of main analysis: (1) 1st percentile of the temperature distribution (0°C) and 1st percentile of the relative humidity distribution (23%); (2) a natural cubic spline with 3° of freedom to model the effect of temperature and relative humidity; and (3) maximum lag period of 24 h.

Analyses using different exposure thresholds showed stable associations with cold temperature. Even when shifting the cutoff from the 1st percentile (main analysis: OR = 5.42, 95% CI: 2.49–11.78) to the 2.5th (0.7 °C, OR = 5.31, 95% CI: 2.51–11.22), 5th (2.0 °C, OR = 5.19, 95% CI: 2.52–10.70), 10th (4.0 °C, OR = 4.96, 95% CI: 2.50–9.81), and 15th percentiles (5.6 °C, OR = 4.71, 95% CI: 2.44–9.06), the risk remained statistically significant, supporting the robustness of the cold exposure effect.

In contrast, the association between low relative humidity and ICH appeared more sensitive to modeling parameters. Although significant in the main model (OR = 1.43, 95% CI: 1.05–1.94) and after removing air pollutant covariates (OR = 1.41, 95% CI: 1.05–1.88), the association lost statistical significance when the lag period was extended to 36 h (OR = 1.29, 95% CI: 0.98–1.69) or 48 h (OR = 1.22, 95% CI: 0.95–1.56), or when extreme humidity values were retained (OR = 1.21, 95% CI: 0.94–1.57). Furthermore, the effect was only significant at the lowest humidity percentile (1st or 2.5th percentile), but diminished at higher percentiles, indicating a possible threshold effect rather than a linear association.

These findings affirm the robustness of the cold temperature-ICH relationship and suggest that extremely low humidity may also act as a short-term environmental trigger, albeit in a more threshold- and time-dependent manner.

## Discussion

Our findings demonstrate a pronounced and temporally distinct association between extreme low temperature, low relative humidity, and ICH, with the cold effect exhibiting a delayed escalation over 0–24 h lag period. The lag effect for temperature became insignificant after 16 h. This delayed risk pattern aligns with sustained hemodynamic strain, where prolonged vasoconstriction and blood pressure variability—amplified by cold-induced sympathetic activation—may gradually increase cerebrovascular shear stress, culminating in vessel rupture.^15^^,^^16^ Complementing this, cold exposure elevates fibrinogen levels and platelet aggregation, exacerbating microvascular permeability over time. In contrast, low humidity (1st percentile: 23%) showed transient effects, with risk peaking earlier at lag 3 h and diminishing rapidly, though remaining significant at 24 h. This temporal mismatch suggests divergent mechanistic pathways: cold exposure may lead to cumulative hemodynamic and hemostatic disturbances, increasing microvascular permeability, and potentially contributing to cerebrovascular events.^17^ In contrast, low humidity may transiently affect systemic inflammation, which could influence cerebral microvascular function.^18^ However, further research is needed to elucidate the specific mechanisms by which these environmental factors impact cerebral microvascular permeability and cerebrovascular health.

Several biological mechanisms may underline the observed associations between environmental conditions and acute ICH onset.

(1) Cold exposure may trigger sympathetic nervous system activation, leading to vasoconstriction and increased systemic blood pressure, thereby elevating shear stress on fragile cerebral vasculature. This process is further compounded by cold-induced elevation in hematocrit, fibrinogen concentration, and platelet aggregability, all of which contribute to a hypercoagulable state and increased risk of vascular rupture, especially in structurally compromised vessels.^15^^,^^16^^,^^19^
(2) Low humidity, independent of temperature, has been associated with increased systemic inflammation and oxidative stress, which may impair endothelial function and disrupt blood-brain barrier integrity. Dehydration resulting from dry air may also increase blood viscosity, further elevating cerebrovascular stress.^20^^,^^21^^,^^22^
(3) Although our findings did not indicate a statistically significant temperature-humidity interaction, it is biologically plausible that simultaneous exposure to cold and dry environments could amplify vascular vulnerability through additive hemodynamic strain and inflammatory pathways. Future experimental studies are warranted to test this hypothesis.^6^^,^^23^

Subgroup analyses further identified lobar hemorrhage patients as particularly vulnerable to cold exposure, a magnitude exceeding risks for deep or infratentorial hemorrhage. This susceptibility likely stems from the pathological fragility of amyloid-laden vasculature in cerebral amyloid angiopathy (CAA), which may render these vessels less adaptive to pressure fluctuations. Notably, smokers exhibited attenuated cold effects, possibly due to nicotine-induced vascular tolerance from chronic α-adrenergic stimulation. Moreover, the COVID-19 pandemic may have introduced shifts in health-seeking behaviors and indoor microclimates (e.g., prolonged indoor stay and altered heating/humidity conditions), which could modify cold and dryness exposures. Future research that accounts for lockdown periods, changes in mobility patterns, and indoor environmental conditions could clarify the interplay between pandemic-related behaviors and environmental stroke triggers.

Sensitivity analyses confirmed the stability of the model: extending the maximum lag period to 48 h reduced effect sizes but maintained significance, confirming cold’s prolonged ecological impact. Adjustment for air pollutants (PM_2.5_, NO2, SO_2_, NO_2_, O_3_, and CO) minimally altered estimates, excluding co-pollutant confounding. Retaining extreme temperature values amplified the cold effect, suggesting that conventional outlier exclusion in meteorological studies may underestimate risks. When contextualized against prior research, the stronger cold effects observed here may reflect the subtropical population’s reduced cold acclimatization and the analytic capture of delayed hemodynamic cascades through hourly lag modeling.

Consistent with the notion of “short-term displacement” described by Bhaskaran et al.^24^ and supported by He et al.,^25^ our data suggest a harvesting phenomenon for both cold and dryness. In essence, the immediate spike in ICH risk among vulnerable individuals is followed by a compensatory decline in risk as the susceptible pool is reduced. Specifically, we observed that extreme cold exposure substantially increased the risk in the first 12 h, reflecting an acute trigger for those already on the verge of decompensation—after which the risk estimates subsided. A similar though earlier peaking pattern emerged for low humidity, further illustrating that distinct environmental factors may produce similarly concentrated, short-term surges in cerebrovascular events. Recognizing these harvesting effects is crucial for interpreting the observed lag patterns and for shaping public health interventions that focus resources on the high-risk hours following abrupt cold or dryness events. Findings of this case-crossover study suggest that a transient exposure to low temperature could be a factor in the onset of ICH episodes, with the association occurring at the concurrent hour and lasting for up to 16 h. The study is limited by its sample size and geographic scope, which may restrict the generalizability of findings to populations in diverse climatic regions. Additionally, unmeasured behavioral confounding factors (e.g., individual cold-avoidance behaviors and indoor heating practices) could influence exposure classification accuracy. Furthermore, the interplay between cold and humidity, though hypothesized to synergistically elevate risk, remains untested. Nevertheless, the results emphasize actionable public health measures: cold exposure warrants 24-h risk alerts, particularly for elderly populations and those with suspected CAA, while low humidity interventions—such as morning hydration and indoor humidification—should prioritize acute exposure windows (0–3 h). By delineating environmental hazards through lag-response dynamics and subgroup vulnerabilities, this work advances targeted prevention strategies for climate-sensitive cerebrovascular disease.

### Limitations of the study

Several limitations should be acknowledged. First, exposure assessment was based on data from the nearest outdoor meteorological station to each patient’s residential address. This may have introduced exposure misclassification, particularly for indoor environments, where individuals spend most of their time. Second, we lacked individual-level data on time-activity patterns, air conditioning or heating use, and indoor air quality, all of which could influence actual exposure levels. Third, although we adjusted for air pollutants and used a time-stratified case-crossover design to control for time-invariant confounders, residual confounding from unmeasured variables (e.g., physical activity, infection, or medication use) cannot be fully excluded. Finally, our findings were based on three hospitals in a single city, which may limit generalizability to other regions or climates.

## Resource availability

### Lead contact

Further information and requests for resources and data should be directed to and will be fulfilled by the lead contact, Dr. Kuanming Huang (hkm1111@sina.com).

### Materials availability

The study did not generate new materials.

### Data and code availability

• The anonymized ICH patient dataset generated and analyzed in this study has been deposited in Mendeley Data and is publicly available at Mendeley Data (https://doi.org/10.17632/7t35pw9wsy.1).
• The R code used for statistical analysis is provided in the supplementary files (Data S1) of this article.
• Meteorological data and air pollution data are available from the China Meteorological Data Service Center (http://data.cma.cn).

## Acknowledgments

This study was financially supported by the 10.13039/100017958Health Commission of Hubei Province, China (no. WJ2023M165) and Hubei 10.13039/501100016983Clinical Research Center of Central Nervous System Repair and Functional Reconstruction, China (no. 2025SJZX011). They did not influence the study’s design, data gathering or analysis, publication decision, or manuscript preparation. We sincerely thank Dr. J.Z. for developing various models for data analysis in this article and extend our gratitude to all the hospitals that provided data.

## Author contributions

S.H. conceived the study, designed the research, conducted the data analysis, prepared the figures, and drafted the manuscript. H.P. contributed to the study design, data analysis, and manuscript drafting. Y.X. performed the formal analysis and contributed to data curation. J.Z. supervised the project, provided resources, and participated in methodological development and validation. Z.W., A.Z., C.H., Y.S., and Y.T. were responsible for data collection and organization. K.H. supervised the overall study, acquired funding, contributed to visualization and methodology, and participated in drafting, reviewing, and editing the manuscript.

## Declaration of interests

The authors declare no competing interests.

## Declaration of generative AI and AI-assisted technologies in the writing process

During the preparation of this work, the authors used ChatGPT and Deep Seek in order to enhance the readability and clarity of this manuscript. The AI assistance was limited to language editing and did not contribute to the research design, data analysis, interpretation, or authorship of the content. After using this tool, the authors reviewed and edited the content as needed and take full responsibility for the content of the publication.

## STAR★Methods

### Key resources table

**Table:** 

REAGENT or RESOURCE	SOURCE	IDENTIFIER
**Deposited Data**		

Meteorological data (temperature, humidity)	China Meteorological Data Service Center	http://data.cma.cn
Air pollution data (PM2.5, NO2, etc.)	China National Environmental Monitoring Center	https://air.cnemc.cn:18007
ICH patient clinical data	Peng, Hao (2025), “ICH Patient Dataset for Case-Crossover Analysis of Temperature and Humidity Effects”, Mendeley Data, V1, https://doi.org/10.17632/7t35pw9wsy.1	https://doi.org/10.17632/7t35pw9wsy.1

**Software and Algorithms**		

R version 4.4.2	R Foundation	https://www.r-project.org
dlnm package (v2.4.7)	Gasparrini et al.^26^	https://cran.r-project.org/web/packages/dlnm
survival package (v3.7-0)	R Foundation	https://cran.r-project.org/web/packages/survival
splines package (v4.4.2)	R Foundation	https://stat.ethz.ch/R-manual/R-devel/library/splines/html/00Index.html
tsModel package (v0.6-2)	R Foundation	https://cran.r-project.org/web/packages/tsModel
forecast package (v8.24.0)	R Foundation	https://cran.r-project.org/web/packages/forecast
dplyr, tidyr packages (v1.1.4, 1.3.1)	Tidyverse/R Foundation	https://www.tidyverse.org/
epiDisplay package (v3.5.0.2)	R Foundation	https://cran.r-project.org/web/packages/epiDisplay
lubridate package (v1.9.4)	R Foundation	https://cran.r-project.org/web/packages/lubridate

### Experimental model and study participant details

The northwestern region of Hubei Province lies between 32°00′ and 33°00′ N latitude and 110°00′ and 111°00′ E longitude. This area experiences a humid subtropical monsoon climate (Köppen classification Cfa) with four distinct seasons, characterized by hot and humid summers and relatively cold, moist winters. Most precipitation occurs during the summer months, and the average temperature during the study period was 16.8°C. Given these climatic characteristics, the region offers valuable insights for research on other mid-latitude mountainous monsoon areas. The study was reviewed and approved by the Ethics Committee of Hubei University of Medicine. Informed consent was waived by the ethics committee because only de-identified, retrospective patient data were analyzed.

All patients were admitted to three stroke units from three large 3A (Class Three/Grade A) hospitals between January 1, 2019 and December 31, 2024 in Shiyan, China. Cases of ICH were retrospectively analyzed by two neurosurgeons according to the International Classification of Diseases, 10th Revision (I61-I62) criteria. For this analysis, we included patients with acute ICH who met the following criteria: (1) age 18 years or older; (2) All patients with ICH underwent CT or MRI evaluation within 24 hours of symptom onset. Patients diagnosed with primary intraventricular hemorrhage or ICH secondary to vascular abnormalities, trauma, brain tumors, hemorrhagic transformation of cerebral infarction, hematological disorders, coagulation dysfunction, or any other suspected secondary causes were excluded from the study. (3) Patients admitted more than 72 hours after symptom onset were excluded from the study to minimize errors attributable to recall bias. (4) All patients were long-term residents of the northwestern region of Hubei Province.

Data on patient demographics (e.g., age and sex), diagnoses, the date and time of symptom onset, medical history, and clinical examination results were collected. The 24-hour day was divided into six intervals (0–3, 4–7, 8–11, 12–15, 16–19, and 20–23 hours) to observe the distribution of onset times in hemorrhagic stroke patients. ICH location was determined based on the first available CT or MRI scan, and patients with an indeterminate hemorrhage location were excluded. Deep ICH was defined as a hematoma confined exclusively to the basal ganglia or thalamus, lobar ICH as a hematoma originating from the cortex and/or the cortical–subcortical junction, and infratentorial ICH as a hematoma involving the cerebellum and/or brainstem. Additionally, all patients underwent a GCS evaluation upon their initial hospital admission. The GCS is a standardized neurological assessment tool used to evaluate a patient’s level of consciousness after a brain injury or acute neurological event (e.g., stroke, trauma). It assesses three categories of responses: eye opening, verbal response, and motor response. Each category is scored individually, and the total score ranges from 3 (deep coma) to 15 (fully conscious).

### Method details

#### Study design

In this case-crossover design, only ICH patients were included, with each individual serving as their own control. This approach inherently minimizes confounding from both measured and unmeasured factors that remain constant within an individual over time, this design effectively controls confounding factors that are time-invariant or remain stable over short periods, such as comorbidities, demographic characteristics, and behavioral risk factors. This method adjusts for long-term trends and seasonal fluctuations in baseline ICH incidence, mitigates time-related biases in the exposure, and controls for confounding factors that remain constant over time,^27^ such as individual risk factors including hypertension, diabetes, smoking, alcohol consumption, hypercholesterolemia, obesity, and a history of cardiovascular disease. The hour of self-reported acute ICH onset is designated as the case hour. For each case hour, three or four control hours are selected that match the same attributes, including year, month, day of the week, and hour of the day. This matching process controls long-term trends, seasonality, and circadian variations. For example, if the initial ICH symptom occurred at 10:00 a.m. on Friday, October 8, 2021, then 10:00 a.m. on all other Fridays in October 2021 (October 1, 15, and 29) would be defined as the control hours (Figure 1).

#### Exposure data

We obtained the data on hourly temperature and relative humidity from the nearest weather stations affiliated with the China Meteorological Data Sharing Service System. There are 7 weather stations included in the analysis, namely Zhushan (ID: 57257), Zhuxi (ID: 57249), Shiyan (ID: 57256), Danjiangkou (ID: 57260), Fangxian (ID: 57259), Yunxi (ID: 57251), and Yunyang (ID: 57253). All stations are part of the China Meteorological Data Sharing Service System, a national platform for authoritative meteorological data. We excluded hourly temperature values below the 1st percentile or above the 99th percentile to minimize the impact of extreme values. Hourly meteorological data from the nearest weather station were matched to each patient based on their residential address and only addresses located within a 50 km radius of a weather station were included to reduce exposure measurement error. We further collected hourly concentrations of fine particulate matter, nitrogen dioxide, sulfur dioxide, ozone, and carbon monoxide from the nearest monitoring stations via the National Urban Air Quality Real-Time Publishing Platform.

#### Sensitivity analyses

The sensitivity analysis involved altering key aspects of the model to assess the robustness of our findings. First, we adjusted the degrees of freedom for the natural cubic splines from 3 to 4 in the exposure dimension to test the stability of the exposure-response curves. Second, we extended the maximum lag window from 24 hours to 36 and 48 hours to determine if the associations remained stable across different time frames. Lastly, we examined various percentile thresholds for low temperature and humidity to assess the dose-response relationship. These adjustments consistently showed that our core conclusions remained stable supporting the robustness of our model and confirming the significant impact of cold and dry conditions on ICH risk.

### Quantification and statistical analysis

We used a bi-dimensional DLNM^26^ within a time-stratified case-crossover design to assess how temperature and relative humidity affect ICH risk. Specifically, we constructed separate cross-basis functions for temperature and humidity, using natural splines in the exposure dimension (the range of temperature or humidity values) to model the non-linear exposure–response relationship, and applying logarithmically spaced knots in the lag dimension (0–24 hours) to capture delayed effects across different time intervals. These cross-basis functions were then incorporated into a conditional logistic regression model, which also included other potential confounders such as air pollutants and temporal trends. Finally, using the crosspred function in dlnm package, we visualized the changes in ICH risk across the two-dimensional “temperature × lag” and “relative humidity × lag” spaces, providing richer and more intuitive evidence for assessing the potential impact of meteorological conditions on ICH risk. The 99th percentile of temperature and relative humidity were used as the reference throughout the study. Based on existing evidence and underlying mechanisms, the 24-hour model can effectively capture the immediate risk of sudden temperature drops. Therefore, we selected *a priori* a maximum lag of 24 hours in the distributed lag nonlinear model. We depicted the lag pattern of the ORs by comparing extremely low temperatures and relative humidity (defined as the 99th percentile of the distributions)^5^^,^^6^^,^^28^ with the reference. Visualizing these lag patterns allowed us to identify the specific lag period associated with the highest risk, which was subsequently used to estimate cumulative associations and plot the exposure-response curves, using the 99th percentile as the reference. After excluding data below the 1st percentile and above the 99th percentile, the 1st percentile of the temperature and humidity distribution was defined as extremely low temperature and extremely low relative humidity, while the 99th percentile was designated as the reference for comparison. We then computed the ORs and 95% CIs for ICH by comparing the extremely low temperature and relative humidity with the reference.

We performed stratified analyses based on several parameters, including gender (male or female), age (>65 years or 18–65 years), smoking status (yes or no), alcohol consumption (yes or no), GCS score (≤8, 9–11, 12–14, 15), and ICH location (Deep, Infratentorial, Lobar), among others, to explore potential individual-level modifiers of the association. Stratum-specific estimates were compared using z-tests, to further evaluate whether temperature and humidity interact in influencing ICH risk, we incorporated a temperature × humidity interaction term into the DLNM. This additional analysis aimed to assess whether any synergistic effects existed between the two environmental exposures. Based on the corresponding point estimates and their standard errors.^29^ Additionally, 9 sensitivity analyses were conducted to assess the robustness of the findings.

All analyses were performed with R software, version 4.4.2 (R Foundation for Statistical Computing Vienna, Austria).

### Additional resources

This study did not involve clinical trials or human participants. All data were obtained from publicly available meteorological databases and de-identified hospital records. As such, ethical approval and clinical registry were not applicable, and the requirement for ethical approval was waived by the institutional ethics committee.

## References

[1] Feigin V.L., Brainin M., Norrving B., Martins S.O., Pandian J., Lindsay P., F Grupper M., Rautalin I. (2025). World Stroke Organization: Global Stroke Fact Sheet 2025. Int. J. Stroke.

[2] Tu W.-J., Chao B.-H., Yan F., Cao L., Wang L.-D. (2020). Stroke unit care for ischemic stroke in China: results of a nation-based study. Intensive Care Med..

[3] Costello A., Abbas M., Allen A., Ball S., Bell S., Bellamy R., Friel S., Groce N., Johnson A., Kett M. (2009). Managing the health effects of climate change. Lancet.

[4] B A., H K., M K., D R., Si P., Am V.-C., Y G., E L., B A., F S. (2024). Extreme Temperatures and Stroke Mortality: Evidence From a Multi-Country Analysis. Stroke.

[5] Guo Y., Barnett A.G., Pan X., Yu W., Tong S. (2011). The Impact of Temperature on Mortality in Tianjin, China: A Case-Crossover Design with a Distributed Lag Nonlinear Model. Environ. Health Perspect..

[6] Alahmad B., Khraishah H., Royé D., Vicedo-Cabrera A.M., Guo Y., Papatheodorou S.I., Achilleos S., Acquaotta F., Armstrong B., Bell M.L. (2023). Associations Between Extreme Temperatures and Cardiovascular Cause-Specific Mortality: Results From 27 Countries. Circulation.

[7] Salam A., Kamran S., Bibi R., Korashy H.M., Parray A., Mannai A.A., Ansari A.A., Kanikicharla K.K., Gashi A.Z., Shuaib A. (2019). Meteorological Factors and Seasonal Stroke Rates: A Four-year Comprehensive Study. J. Stroke Cerebrovasc. Dis..

[8] Rakers F., Schiffner R., Rupprecht S., Brandstädt A., Witte O.W., Walther M., Schlattmann P., Schwab M. (2016). Rapid weather changes are associated with increased ischemic stroke risk: a case-crossover study. Eur. J. Epidemiol..

[9] Zhu X., Chen R., Yuan J., Liu Y., Wang Y., Ji X., Kan H., Zhao J. (2024). Hourly Heat Exposure and Acute Ischemic Stroke. JAMA Netw. Open.

[10] Woo D., Comeau M.E., Venema S.U., Anderson C.D., Flaherty M., Testai F., Kittner S., Frankel M., James M.L., Sung G. (2022). Risk Factors Associated With Mortality and Neurologic Disability After Intracerebral Hemorrhage in a Racially and Ethnically Diverse Cohort. JAMA Netw. Open.

[11] Chen S.-J., Lee M., Wu B.-C., Muo C.-H., Sung F.-C., Chen P.-C. (2024). Meteorological factors and risk of ischemic stroke, intracranial hemorrhage, and subarachnoid hemorrhage: A time-stratified case-crossover study. Int. J. Stroke.

[12] Liu P., Chen Z., Xia X., Wang L., Li X. (2023). Potential role of ambient temperature as a trigger for intracerebral hemorrhage: a time-stratified case-crossover study in Tianjin, China. Environ. Sci. Pollut. Res..

[13] Lavados P.M., Olavarría V.V., Hoffmeister L. (2018). Ambient Temperature and Stroke Risk: Evidence Supporting a Short-Term Effect at a Population Level From Acute Environmental Exposures. Stroke.

[14] Herweh C., Nordlohne S., Sykora M., Uhlmann L., Bendszus M., Steiner T. (2017). Climatic and Seasonal Circumstances of Hypertensive Intracerebral Hemorrhage in a Worldwide Cohort. Stroke.

[15] Aubinière-Robb L., Jeemon P., Hastie C.E., Patel R.K., McCallum L., Morrison D., Walters M., Dawson J., Sloan W., Muir S. (2013). Blood Pressure Response to Patterns of Weather Fluctuations and Effect on Mortality. Hypertension.

[16] Fang C.-W., Ma M.-C., Lin H.-J., Chen C.-H. (2012). Ambient temperature and spontaneous intracerebral haemorrhage: a cross-sectional analysis in Tainan, Taiwan. BMJ Open.

[17] Guo Y., Wei C., Ding H., Li P., Gao Y., Zhong K., Bao Z., Qu Z., Wang B., Hu J. (2024). Effects of cold stress on the blood-brain barrier in Plectropomus leopardus. BMC Genom..

[18] Fruekilde S.K., Bailey C.J., Lambertsen K.L., Clausen B.H., Carlsen J., Xu N.L., Drasbek K.R., Gutiérrez-Jiménez E. (2022). Disturbed microcirculation and hyperaemic response in a murine model of systemic inflammation. J. Cereb. Blood Flow Metab..

[19] Greaney J.L., Kenney W.L., Alexander L.M. (2017). Neurovascular mechanisms underlying augmented cold-induced reflex cutaneous vasoconstriction in human hypertension. J. Physiol..

[20] Zhang R., Liu G., Jiang Y., Li G., Pan Y., Wang Y., Wei Z., Wang J., Wang Y. (2018). Acute Effects of Particulate Air Pollution on Ischemic Stroke and Hemorrhagic Stroke Mortality. Front. Neurol..

[21] Guo M., Zhou M., Li B., Du C., Yao R., Wang L., Yang X., Yu W. (2022). Reducing indoor relative humidity can improve the circulation and cardiorespiratory health of older people in a cold environment: A field trial conducted in Chongqing, China. Sci. Total Environ..

[22] Slatina E., Music M., Babic N., Dervisevic A., Mujaric E., Salibasic M., Tuna E., Corovic J. (2013). Correlation Between Change in Air Humidity and the Incidence of Stroke. Mater. Sociomed..

[23] Chen Z., Liu P., Xia X., Wang L., Li X. (2022). The underlying mechanisms of cold exposure-induced ischemic stroke. Sci. Total Environ..

[24] Bhaskaran K., Gasparrini A., Hajat S., Smeeth L., Armstrong B. (2013). Time series regression studies in environmental epidemiology. Int. J. Epidemiol..

[25] He C., Breitner S., Zhang S., Huber V., Naumann M., Traidl-Hoffmann C., Hammel G., Peters A., Ertl M., Schneider A. (2024). Nocturnal heat exposure and stroke risk. Eur. Heart J..

[26] Gasparrini A. (2011). Distributed Lag Linear and Non-Linear Models in R: The Package dlnm. J. Stat. Softw..

[27] Chen K., Breitner S., Wolf K., Hampel R., Meisinger C., Heier M., Von Scheidt W., Kuch B., Peters A., Schneider A., KORA Study Group (2019). Temporal variations in the triggering of myocardial infarction by air temperature in Augsburg, Germany, 1987–2014. Eur. Heart J..

[28] Zhang H., Wang Q., Benmarhnia T., Jalaludin B., Shen X., Yu Z., Ren M., Liang Q., Wang J., Ma W., Huang C. (2021). Assessing the effects of non-optimal temperature on risk of gestational diabetes mellitus in a cohort of pregnant women in Guangzhou, China. Environ. Int..

[29] Liu Y., Pan J., Fan C., Xu R., Wang Y., Xu C., Xie S., Zhang H., Cui X., Peng Z. (2021). Short-Term Exposure to Ambient Air Pollution and Mortality From Myocardial Infarction. J. Am. Coll. Cardiol..

