# Supplementary material for: Extreme temperature and humidity exposure elevates acute intracerebral hemorrhage risk
Source: iScience. 2025 Nov 5;29(1):113956. doi: 10.1016/j.isci.2025.113956 (PMC12799769; doi:10.1016/j.isci.2025.113956)
Supplement: Document S1. Figures S1–S5 and Data S1 [file mmc1.pdf]

iScience, Volume *29*

Supplemental information

Extreme temperature and humidity exposure elevates acute intracerebral hemorrhage risk

Shengli Hu, Hao Peng, Yong Xu, Jun Zhao, Zhizhen Wei, Anwei Zhang, Can Huang, Yuting Si, Yingying Tang, and Kuanming Huang

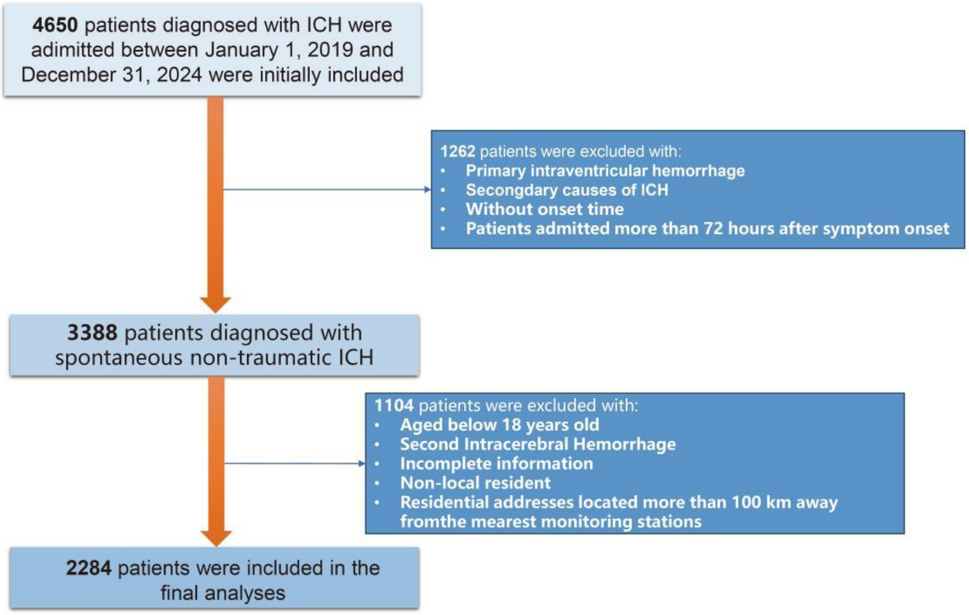

Figure S1. Flowchart for the Inclusion and Exclusion of ICH Patients.

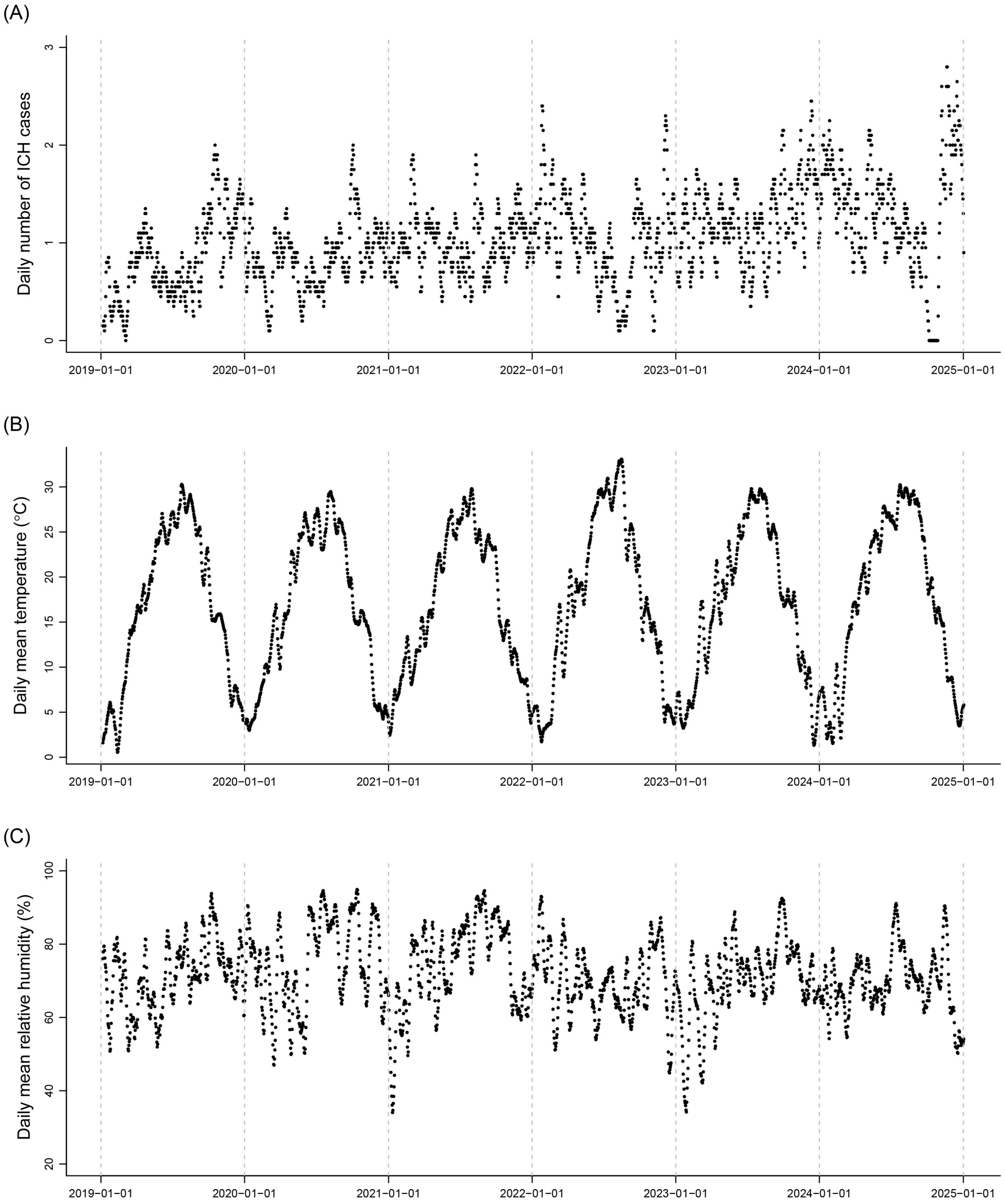

Figure S2. 10-day moving Average Trends of ICH Cases, Temperature, and Humidity.

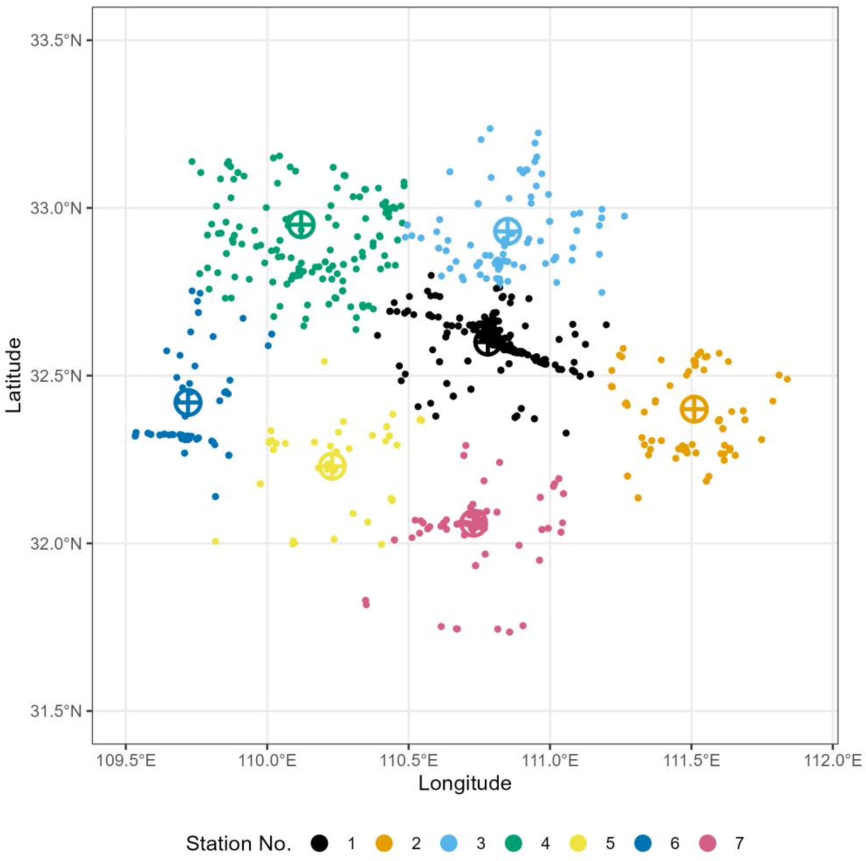

Figure S3. Geographic distribution of residential addresses of ICH patients and corresponding nearest meteorological stations. Station 1: Shiyan (ID: 57256), Station 2: Danjiangkou (ID: 57260), Station 3: Yunyang (ID: 57253), Station 4: Yunxi (ID: 57251), Station 5: Zhuxi (ID: 57249), Station 6: Zhushan (ID: 57257), and Station 7: Fangxian (ID: 57259).

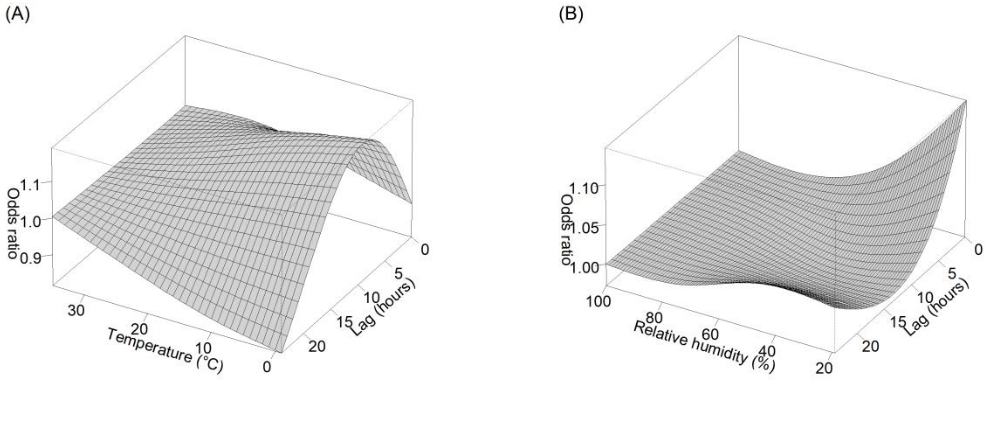

Figure S4. The overall effect of temperature and relative humidity 3D plot.

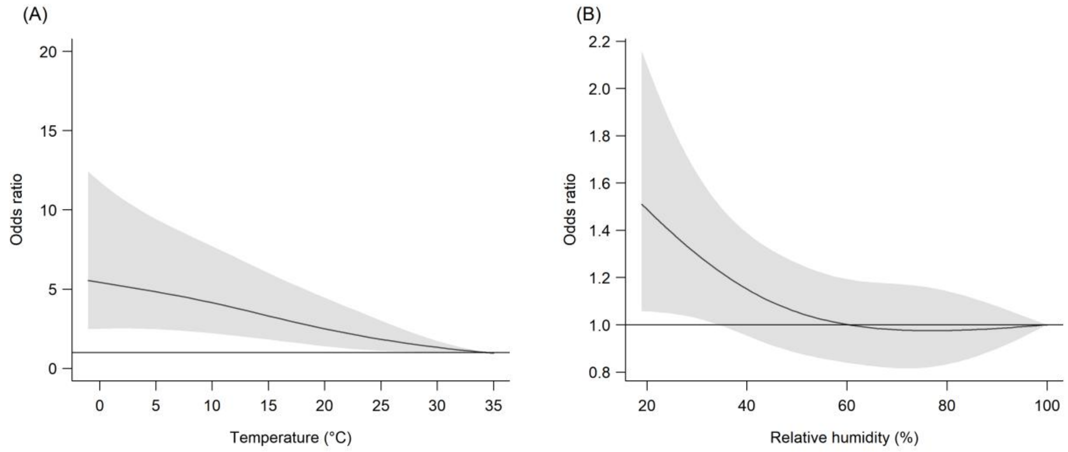

Figure S5. Temperature 16-hour and RHU 3-hour effect plot.

Data S1 / Methods S1: R code for case-crossover and DLNM analysis of acute intracerebral hemorrhage risk

```
library(dplyr)
library(tidyr)
library(tsModel)
library(dlnm)
library(forecast)
library(survival)
library(splines)
library(lubridate)

ICH_data <- read.csv("data/allpatient.csv")
weather_data <- read.csv("data/alldata.csv")
aq_data <- read.csv("data/allaqdata.csv")

meteo_data <- merge(ICH_data, weather_data, by = c("localDate", "Hour"), all = TRUE)
case_counts <- ICH_data %>%
  filter(case == 1) %>%
  group_by(date_time) %>%
  summarise(weights = n(), .groups = 'drop')

dat <- meteo_data %>%
  left_join(case_counts, by = "date_time") %>%
  tidyr::replace_na(list(weights = 1)) %>%
  distinct(date_time, .keep_all = TRUE) %>%
  group_by(stratum) %>%
  mutate(weights = max(weights)) %>%
  ungroup() load("data/merged.rdata")
```

```
# Exclude hourly temperatures below the first percentile or above
# the 99th percentile to minimize the implications of extreme values
dat1 <- dat %>% filter(TEM >= quantile(TEM, 0.01) & TEM <= quantile(TEM, 0.99)) %>%
  filter(RHU >= quantile(RHU, 0.01) & RHU <= quantile(RHU, 0.99))

dat2 <- dat %>% group_by(localDate) %>%
  mutate(counts = sum(case),
            temp = mean(TEM, na.rm = T),
            rhu = mean(RHU, na.rm = T)) %>%
  ungroup() %>%
  distinct()

plot(ma(dat2$counts, 10), type = "p", pch = 20, bty = "l",
      xlab = "", ylab = "", xaxt = "n", yaxt = "n", ylim = c(0, 3))
axis(1, at = grep("-01-01",dat2$localDate),
      labels = c("2019-01-01", "2020-01-01", "2021-01-01", "2022-01-01", "2023-01-01",
"2024-01-01", "2025-01-01"))
axis(2, at = c(0, 1, 2, 3), labels = 0:3)
abline(v = grep("-01-01",dat2$localDate), lty = 2, col = "grey")
mtext("Daily number of ICH cases", line = 2.5, side = 2)
mtext("(A)", side = 3, adj = -0.07, line = 1, cex = 1.2)

plot(ma(dat2$temp, 10), type = "p", pch = 20, bty = "l",
      xaxt = "n", yaxt = "n", xlab = "", ylab = "")
axis(1, at = grep("-01-01",dat2$localDate),
      labels = c("2019-01-01", "2020-01-01", "2021-01-01", "2022-01-01", "2023-01-01",
"2024-01-01", "2025-01-01"))
axis(2, at = c(0, 5, 10, 15, 20, 25, 30, 35))
abline(v = grep("-01-01",dat2$localDate), lty = 2, col = "grey")
mtext(expression(paste("Daily mean temperature (",degree,"C)")), line = 2.5, side = 2)
mtext("(B)", side = 3, adj = -0.07, line = 1, cex = 1.2)

plot(ma(dat2$rhu, 10), type = "p", pch = 20, ylim = c(20, 100), bty = "l",
      xaxt = "n", yaxt = "n", xlab = "", ylab = "")
axis(1, at = grep("-01-01",dat2$localDate),
      labels = c("2019-01-01", "2020-01-01", "2021-01-01", "2022-01-01", "2023-01-01",
"2024-01-01", "2025-01-01"))
axis(2, at = c(0, 20, 40, 60, 80, 100))
abline(v = grep("-01-01",dat2$localDate), lty = 2, col = "grey")
mtext("Daily mean relative humidity (%)", line = 2.5, side = 2)
mtext("(C)", side = 3, adj = -0.07, line = 1, cex = 1.2)

# Descriptive Statistics of Environmental Data
dat1 %>%
```

```
  filter(case == 1) %>%
  dplyr::select(avg_TEM_24h_before, avg_RHU_24h_before,
                avg_PM2_5_24h_before, avg_PM10_24h_before, avg_SO2_24h_before,
                avg_NO2_24h_before, avg_O3_24h_before, avg_CO_24h_before,
weights) %>%
  uncount(weights) %>%
  pivot_longer(cols = 1:8, names_to = "ind", values_to = "value") %>%
  mutate(ind = factor(ind, levels = c("avg_TEM_24h_before", "avg_RHU_24h_before",
                                      "avg_PM2_5_24h_before",
"avg_PM10_24h_before",
                                      "avg_SO2_24h_before", "avg_NO2_24h_before",
                                      "avg_O3_24h_before", "avg_CO_24h_before"),
                      labels = c("TEM", "RHU", "PM2_5", "PM10", "SO2", "NO2", "O3_8h",
"CO"))) %>%
  group_by(ind) %>%
  summarise('Mean (SD)' = paste0(round(mean(value, na.rm = T),2), " (", round(sd(value,
na.rm = T), 1), ")"),
            P1 = round(quantile(value, 0.01, na.rm = T), 2),
            P25 = round(quantile(value, 0.25, na.rm = T), 2),
            P50 = round(quantile(value, 0.50, na.rm = T), 2),
            P75 = round(quantile(value, 0.75, na.rm = T), 2),
            P99 = round(quantile(value, 0.99, na.rm = T), 2))

# modeling
max_lag <- 24
varknots.TEM <- equalknots(dat1$TEM, fun = "ns", df = 3)
logknots <- logknots(max_lag, fun = "ns", df = 3)
temp.basis <- crossbasis(dat1$TEM, lag = max_lag,
                         argvar = list(knots = varknots.TEM),
                         arglag = list(knots = logknots))

varknots.RHU <- equalknots(dat1$RHU, fun = "ns", df = 3)
rhu.basis <- crossbasis(dat1$RHU, lag = max_lag,
                        argvar = list(knots = varknots.RHU),
                        arglag = list(knots = logknots))

mod1 <- clogit(case ~ temp.basis   + ns(PM2_5, 3) + ns(PM10, 3) +
                 ns(O3_8h, 3) +   ns(SO2, 3) + ns(NO2, 3) + ns(CO, 3) +
                 strata(stratum),
               weights = weights, method = "breslow", data = dat1)
summary(mod1)

mod2 <- clogit(case ~ rhu.basis   + ns(PM2_5, 3) + ns(PM10, 3) +
                 ns(O3_8h, 3) +   ns(SO2, 3) + ns(NO2, 3) + ns(CO, 3) +
```

```
                    strata(stratum),
                  weights = weights, method = "breslow", data = dat1)
summary(mod2)

quantile(dat1$TEM, probs = c(0.01, 0.99))
temp.pred <- crosspred(temp.basis, mod1, cumul = TRUE,
                         cen = quantile(dat1$TEM, probs = 0.99),
                         by = 1, bylag = 1)
quantile(dat1$RHU, probs = c(0.01, 0.99))
rhu.pred <- crosspred(rhu.basis, mod2, cumul = TRUE,
                        cen = quantile(dat1$RHU, 0.99),
                        by = 1, bylag = 1)

plot(temp.pred, "3d", r = 90, d = 0.3,
     font.lab = 1.5, shade = NA, col = "lightgrey",
     xlab = "Temperature (°C)", ylab = "Lag (hours)", zlab = "Odds ratio",
     expand = 0.6, lwd = 0.5)
mtext("(A)", side = 3, adj = -0.02, cex = 1.2)
plot(rhu.pred, "3d",   r = 90, d = 0.3,
     font.lab = 1.5, shade = NA, col = "lightgrey",
     xlab = "Relative humidity (%)",
     ylab = "Lag (hours)",
     zlab = "Odds ratio",
     expand = 0.6, lwd = 0.5)
mtext("(B)", side = 3, adj = -0.02, cex = 1.2)

plot(temp.pred, "overall", font.lab = 1.5, lty = 1, lwd = 2, las = 1,
     xlab = "Temperature (°C)", ylab = "Odds ratio")
mtext("(A)", side = 3, adj = -0.12, line = 0.8, cex = 1.2)
plot(rhu.pred, "overall", font.lab = 1.5, lty = 1, lwd = 2, las = 1,
     xlab = "Relative humidity (%)", ylab = "Odds ratio")
mtext("(B)", side = 3, adj = -0.12, line = 0.8, cex = 1.2)

tablag1 <- with(temp.pred, t(rbind(cumRRfit["0", "lag16"],
                                    cumRRlow["0", "lag16"],
                                    cumRRhigh["0", "lag16"])))
colnames(tablag1) <- c("OR", "ci.low", "ci.hi")
tablag1

tablag2 <- with(rhu.pred, t(rbind(cumRRfit["23", "lag3"],
                                   cumRRlow["23", "lag3"],
                                   cumRRhigh["23", "lag3"])))
colnames(tablag2) <- c("OR", "ci.low", "ci.hi")
tablag2
```

```
df1 <- data.frame(Temperature = rownames(temp.pred$cumRRfit),
                  OR = temp.pred$cumRRfit[, "lag16"],
                  low = temp.pred$cumRRlow[, "lag16"],
                  high = temp.pred$cumRRhigh[, "lag16"])
plot(df1$Temperature, df1$OR, type = "n", bty = "l",
     ylim = c(0,20), las = 1,
     xlab = "Temperature (°C)", ylab = "Odds ratio")
lines(df1$Temperature, df1$OR, lwd = 1.5)
for (i in 1:(nrow(df1) - 1)) {
  polygon(
    c(df1$Temperature[i], df1$Temperature[i+1], df1$Temperature[i+1], df1$Temperature[i]),
    c(df1$low[i], df1$low[i+1], df1$high[i+1], df1$high[i]),
col = rgb(0.7, 0.7, 0.7, alpha = 0.4),
border = NA)
}
abline(h = 1)
mtext("(A)", side = 3, adj = -0.12, line = 0.8, cex = 1.2)
# Relative humidity
df2 <- data.frame(RHU = rownames(rhu.pred$cumRRfit),
                  OR = rhu.pred$cumRRfit[, "lag3"],
                  low = rhu.pred$cumRRlow[, "lag3"],
                  high = rhu.pred$cumRRhigh[, "lag3"])

plot(df2$RHU, df2$OR, type = "n", bty = "l",
     ylim = range(c(df2$low, df2$high)), las = 1,
     xlab = "Relative humidity (%)", ylab = "Odds ratio")
lines(df2$RHU, df2$OR, lwd = 1.5)
for (i in 1:(nrow(df2) - 1)) {
  polygon(
    c(df2$RHU[i], df2$RHU[i+1], df2$RHU[i+1], df2$RHU[i]),
    c(df2$low[i], df2$low[i+1], df2$high[i+1], df2$high[i]),
col = rgb(0.7, 0.7, 0.7, alpha = 0.4),
border = NA)
}
abline(h = 1)
mtext("(B)", side = 3, adj = -0.12, line = 0.8, cex = 1.2)

plot(temp.pred, ptype = "slice", var = 0,   xlab = "Lag (hours)", ylab = "Odds ratio",
     lwd = 1.5, font.lab = 1.5,
     ylim = c(0.55, 1.55), las = 1)
mtext("(A)", side = 3, adj = -0.12, line = 0.8, cex = 1.2)
plot(rhu.pred, var = 23, "slice",
     xlab = "Lag (hours)", ylab = "Odds ratio",
```

```
        lwd = 1.5, font.lab = 1.5,
        ylim = c(0.8, 1.3), las = 1)
mtext("(B)", side = 3, adj = -0.12, line = 0.8, cex = 1.2)

plot(temp.pred, ptype = "slice", var = 0, cumul = TRUE, lwd = 1.5, las = 1, font.lab = 1.5,
        xlab = "Lag (hours)", ylab = "Cumulative odds ratio")
mtext("(A)", side = 3, adj = -0.12, line = 0.8, cex = 1.2)
plot(rhu.pred, var = 23, cumul = TRUE, lwd = 1.5, las = 1, font.lab = 1.5,
        xlab = "Lag (hours)", ylab = "Cumulative odds ratio")
mtext("(B)", side = 3, adj = -0.12, line = 0.8, cex = 1.2)
```
